# Silver Nanoparticles Protect Skin from Ultraviolet B-Induced Damage in Mice

**DOI:** 10.3390/ijms21197082

**Published:** 2020-09-25

**Authors:** Yu-Yi Ho, Der-Shan Sun, Hsin-Hou Chang

**Affiliations:** Department of Molecular Biology and Human Genetics, Tzu-Chi University, Hualien 97004, Taiwan; 104727103@gms.tcu.edu.tw (Y.-Y.H.); dssun@mail.tcu.edu.tw (D.-S.S.)

**Keywords:** silver nanoparticles, titanium dioxide, ultraviolet B, skin damage, sunburn, inflammation, interleukin-1, reactive oxygen species, cell death, sunscreen

## Abstract

Ultraviolet (UV) radiation from sunlight has various adverse effects; thus, UV blockage is recommended for preventing sunburn. Common sunscreen ingredients, such as nanosized titanium dioxide and zinc oxide, offer effective protection and enhance cosmetic appearance; however, health concerns have been raised regarding their photocatalytic activity, which generates reactive oxygen species under UV illumination. Silver nanoparticles (AgNPs) are known as safe materials for use in a wide spectrum of biomedical applications. In vitro studies have revealed that AgNPs may have a protective effect against UV irradiation, but the effects in animal studies remain unclear. The present study demonstrated that AgNPs effectively protect against UVB-induced skin damage both in cell cultures and mouse models. These results suggested that AgNPs are feasible and safe as sunscreen ingredients for protection against UVB-induced skin damage.

## 1. Introduction

Sunlight can be divided into three main categories according to its wavelength: 200–400 nm is ultraviolet (UV), 400–800 nm is visible light, and >800 nm is infrared. UV rays can be further divided into three types by wavelength: 200–280 nm is UVC, 280–320 nm is UVB, and 320–400 nm is UVA [1]. Because shorter UV wavelengths result in greater damage to the human body, the damage caused by UVC is more serious than that of UVB and UVA. However, UVC is largely absorbed by the ozone layer in the atmosphere; thus, UVB is the most critical UV radiation for inducing skin redness and sunburn, pathogenesis involving the induction of reactive oxygen species (ROS), DNA damage, and death of skin cells [1,2,3,4]. The World Health Organization (WHO) developed the UV Index (UVI) for forecasting, reporting, and measuring the extent of UV radiation [5,6].

Experts recommend using sunscreen to block UV radiation and avoiding excessive solar exposure to prevent sunburn [1]. Through mechanisms such as reflection, scattering, and absorption, titanium dioxide (TiO_2_) and zinc oxide (ZnO) can shield and reduce UV irradiation-induced injury [7,8]. TiO_2_ and ZnO nanoparticles (NPs) have been widely used as ingredients in commercial sunscreens since the late 1990s [3]. However, health concerns have been increasing regarding systemic absorption and photocatalytic ROS production [9]. Anatase crystal contamination of nanosized TiO_2_ can elicit photocatalysis and induce cellular damage [10]. Accordingly, a safe and effective substitute for TiO_2_ and ZnO is required.

Silver has been used as a bactericidal agent in hygiene and medicinal applications for thousands of years [11]. Silver nanoparticles (AgNPs) are nontoxic to cultured cells in vitro [12,13,14] and are currently widely used in nanomedicine for managing and cleaning burns, trauma, and diabetic wounds, as well as in catheters, contraceptive devices, dental silver amalgams, and water purification devices [15,16,17,18]. Therefore, AgNPs represent a fast-growing field in nanotechnology-based products [19,20,21]. Bacterial infection is a concern after sunburn [22,23,24]. AgNPs display absorbance in the UV range [25,26] and have strong antibacterial properties [27,28,29]. Thus, AgNPs could be particularly suitable as a sunscreen ingredient. Related studies have primarily focused on feasibility analyses by using in vitro models [12,13,14,30]. The present study revealed that AgNPs can protect skin from UVB-induced damage in both cell cultures and mouse models.

## 2. Results

### 2.1. Shielding Effect of AgNP- and TiO_2_NP-Coated Plastic Films against UVB Irradiation

**Figure 1:**
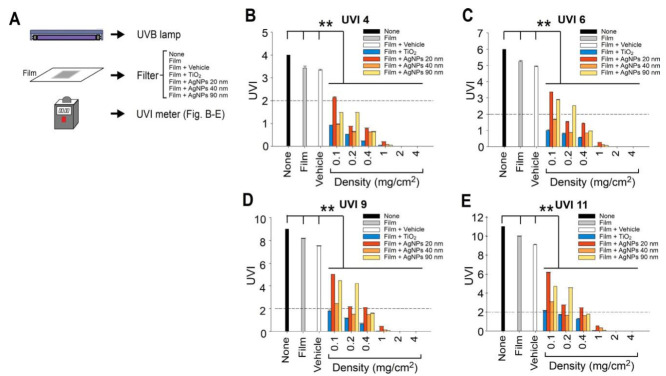
UVB attenuation by TiO_2_NP- and AgNP-coated plastic films. Experiment setting (**A**). Detected UVB levels of the NP-coated films at various NP densities. UVB at UVIs of 4 (100 *M*_W_/m^2^ UVEry) (**B**), 6 (150 *M*_W_/m^2^ UVEry) (**C**), 9 (225 *M*_W_/m^2^ UVEry) (**D**), and 11 (275 *M*_W_/m^2^ UVEry) (**E**). The dashed line indicates a UVI of 2 (50 *M*_W_/m^2^ UVEry). UVB UVI below this level is considered safe (B–E). *n* = 3, ** *p* < 0.001. UVB, ultraviolet B; UVI, ultraviolet index; AgNP, silver nanoparticle; TiO_2_, titanium dioxide. Data are represented as mean ± standard deviation.

Because related studies have suggested that the size of AgNPs is critical for UV protection, we analyzed three different sizes of AgNPs, namely 20, 40, and 90 nm. Based on modified versions of previously reported methods [31], we measured the UVB-protection of 20, 40, and 90 nm AgNPs as well as that of 20 nm TiO_2_NPs (Figure 1A). Although we observed variations in the UVB-shielding effect of AgNPs, all the tested NPs markedly reduced UVB intensity to within a safe range (UV index UVI < 2, dashed lines in Figure 1B–E), as defined by the WHO [5,6]. The effect remained even under extreme UVB exposure of 11 at doses higher than 1 mg/cm^2^ (Figure 1E). At an NP density of 2 mg/cm^2^, TiO_2_NPs and AgNPs exhibited a UVB-blocking efficiency of >99%. More detailed results of the respective NP performance for densities exceeding 1 mg/cm^2^ are available in the Supplementary File (Figure S1). Because 20 and 40 nm AgNPs tend to form aggregates at high densities (>0.4 mg/cm^2^), 90 nm AgNPs were easier to distribute evenly in the vehicle when spreading on the films. This likely explains why, compared with 20 and 40 nm AgNPs, 90 nm AgNPs had a greater UV-shielding effect at high densities (1, 2, and 4 mg/cm^2^) (Supplementary Figure S1). These data suggested that the protection against UVB irradiation provided by AgNPs is effective and comparable to that of TiO_2_NPs.

### 2.2. Shielding Effect of AgNPs Protects Cells from UVB Damage

**Figure 2:**
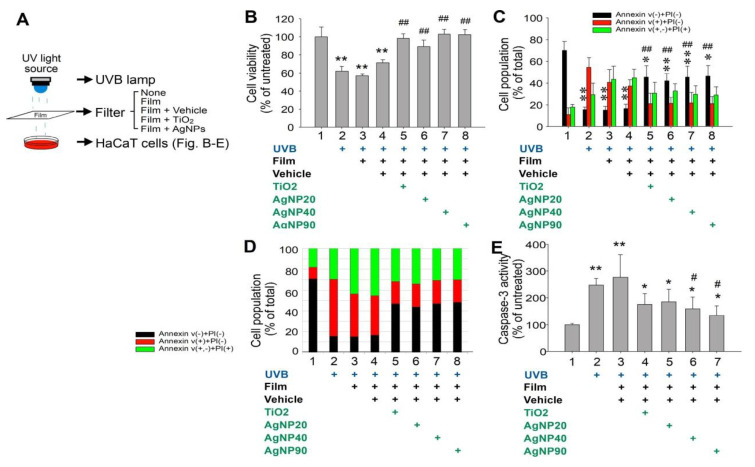
AgNPs protect HaCaT keratinocytes from UVB damage. Experiment setting (**A**). TiO_2_NPs and AgNPs mediated the rescue of UVB-induced cell death among human immortalized keratinocyte HaCaT cells (UVI 6 for 20 min (135.9 mJ/cm^2^) at 1 mg/cm^2^), according to XTT (**B**), flow cytometry (**C**,**D**) (annexin V and propidium iodide (PI) staining), and caspase-3 (**E**) assays. Black columns indicate annexin V^−^/PI^−^ (surviving cells), red columns indicate annexin V^+^/PI^−^ (early stage of cell death), and green columns represent PI^+^ (late stage of cell death). *n* = 6 (three experiments with two replicates). Groups without UVB exposure (UVB (−) untreated control) were normalized to 100% (**B**,**E**). The total cell population of each group was normalized to 100% (**C**,**D**). *n* = 6 (three experiments with two replicates). * *p* < 0.05, ** *p* < 0.01 vs. UVB (−) groups; # *p* < 0.05, ## *p* < 0.01 vs. vehicle groups. UVB, ultraviolet B; AgNP, silver nanoparticle; TiO_2_, titanium dioxide. Data are shown as mean ± standard deviation.

The protective efficacy of AgNPs was further investigated using a cell culture model. All four NPs (20, 40, and 90 nm AgNPs and TiO_2_NPs) protected human immortalized HaCaT keratinocytes from UVB-induced damage at a density of 1 mg/cm^2^ (Figure 2). After UVB irradiation at a UVI of 6 for 10 min, all four NPs offered considerable protection in human epidermal keratinocyte (HaCaT) cells, as indicated by the mitigation of UV-induced cell death (Figure 2). The data indicated that, because of the shielding effect of AgNPs under UVB irradiation, cell viability was protected (Figure 2A,B). The viable cell population in the group protected with AgNPs was higher than that in groups without NP shielding (Figure 2C,D). Furthermore, the UVB-induced elevation of cell death (Figure 2C–E) was ameliorated.

### 2.3. Shielding Effect of AgNPs Protects Skin from UVB-Induced Elevations of Cellular ROS and Proinflammatory Cytokines

**Figure 3:**
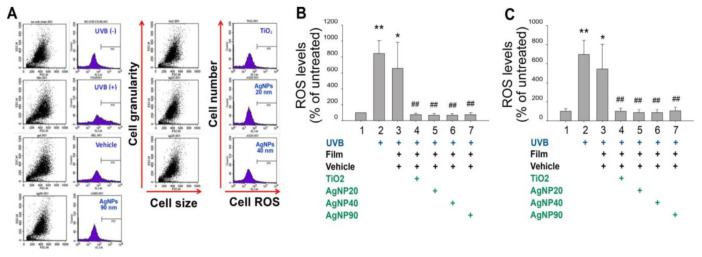
AgNPs rescue UVB-mediated induction of high cellular ROS levels in HaCaT keratinocytes. Flow cytometry (**A**,**B**) and fluorescence microplate reader (**C**) analyses of relative HaCaT cellular ROS levels after UVB irradiation using dichloro-dihydro-fluorescein diacetate (DCFH–DA) labeling. UVB, ultraviolet B; AgNP, silver nanoparticle; TiO_2_, titanium dioxide; ROS, reactive oxygen species. * *p* < 0.05, ** *p* < 0.01 vs. UVB (−) groups; ## *p* < 0.01 vs. vehicle groups.

Cellular ROS production is involved in UV-induced keratinocyte cell death and skin damage [32,33]. We further investigated whether the shielding effect of AgNPs also protected HaCaT cells from UVB-induced elevation of cellular ROS. Using flow cytometry, we observed that AgNP-coated films markedly reduced UVB-induced ROS production in HaCaT cells (Figure 3).

**Figure 4:**
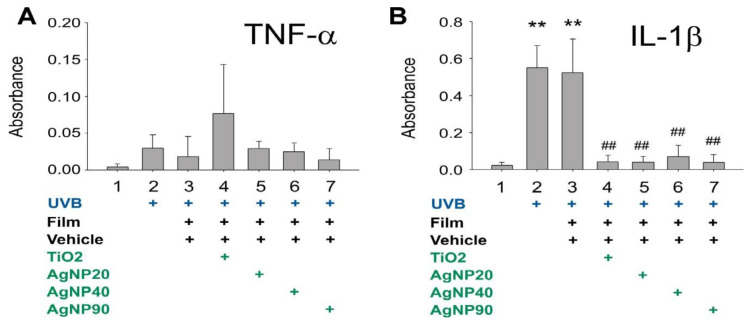
AgNPs rescue UVB-induced releases of proinflammatory cytokines in HaCaT keratinocyte culture. Enzyme-linked immunosorbent assay for the UVB-induced release of the proinflammatory cytokines TNF–α (**A**) and IL–1β (**B**) in a HaCaT cell culture. ** *p* < 0.01 vs. UVB (−) groups; *p* < 0.05, ## *p* < 0.01 vs. UVB (+) and vehicle groups. UVB, ultraviolet B; AgNP, silver nanoparticle; TiO_2_, titanium dioxide; TNF–α, tumor necrosis factor-α; IL–1β, interleukin–1β.

In addition to the elevation of cellular ROS levels, proinflammatory cytokine release is also considered a pathogenic response that exacerbates UVB-induced skin damage [31]. An enzyme-linked immunosorbent assay (ELISA) was conducted to detect elevated tumor necrosis factor-α (TNF-α) and interleukin-1β (IL-1β). These two proinflammatory cytokines are associated with the severity of UVB-induced skin damage [31]. The results revealed that the shielding effect of AgNPs tended to reduce TNF-α release and markedly ameliorated IL-1β release in a HaCaT cell culture (Figure 4).

### 2.4. Shielding Effect of AgNPs Ameliorates UVB-Induced Skin Hyperplasia and Cell Damage in Mice

**Figure 5:**
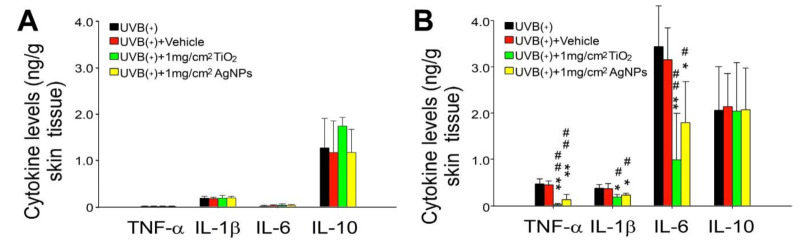
AgNPs rescue UVB-induced skin expression of proinflammatory cytokines in mice. Enzyme-linked immunosorbent assay was performed to examine the expression of proinflammatory cytokines TNF–α, IL–1β, and IL–6 and anti-inflammatory cytokine IL–10 in mouse skin without (**A**) and with (**B**) UVB irradiation. ** *p* < 0.01 vs. UVB (−) groups; # *p* < 0.05, ## *p* < 0.01 vs. vehicle groups. In the UVB-irradiated groups, skin samples were collected 3 days after UVB irradiation (UVI 6, 20 min per day, three cycles, (135.9 mJ/cm^2^/day × 3 days)), with or without protection of 1 mg/cm^2^ NPs. *n* = 6 (three mice in each experiment and repeated twice). * *p* < 0.05, ** *p* < 0.01 vs. the respective UVB (+) and vehicle groups; # *p* < 0.05, ## *p* < 0.01 vs. the respective vehicle groups. UVB, ultraviolet B; AgNP, silver nanoparticle; TiO_2_, titanium dioxide; TNF–α, tumor necrosis factor–α; IL–1β, interleukin–1β; IL–6, interleukin 6; IL–10, interleukin 10. Data are presented as mean ± standard deviation.

To further investigate the protective effect of AgNPs in vivo, we used ELISA to analyze the UVB exposure-induced expression of proinflammatory cytokines, namely TNF–α, IL–6, and IL–1β, as well as that of anti-inflammatory cytokine IL–10 in mouse skin. Because, relative to 20 and 40 nm AgNPs, 90 nm AgNPs are less prone to form aggregates and have superior UV-shielding properties at high densities (>0.4 mg/cm^2^), we employed 90 nm AgNPs for the following in vivo experiments. We performed control experiments using direct treatment of these NPs, without UVB irradiation, to examine whether the treatment alone would lead to sufficient differential expression of the tested cytokines. The results indicated that the treatment of these NPs did not change the basal level expression of the aforementioned cytokines (Figure 5A, Supplementary Figure S2). UVB experiments revealed that both TiO_2_NPs and AgNPs have comparable protective effects against the expression of UVB-induced proinflammatory cytokines TNF–α, IL–6, and IL–1β. By contrast, the expression of anti-inflammatory cytokine IL–10 was not affected by UVB and NPs treatments (Figure 5B).

**Figure 6:**
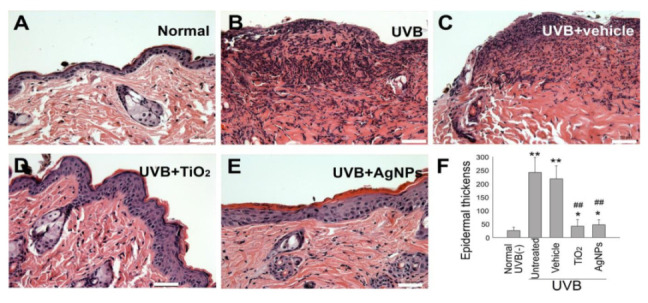
TiO_2_NPs and AgNPs ameliorate UVB-induced skin hyperplasia in mice. Histological examinations of the epidermis and dermis (**A**–**E**) revealed alterations before (**A**) and 3 days after UVB irradiation (UVI 6, 20 min/day, three cycles, (135.9 mJ/cm^2^/day × 3 days)), with and without protection using 1-mg/cm^2^ TiO_2_NPs and 90-nm AgNPs (**B**–**E**) (hematoxylin and eosin staining, × 200; scale bars in (**A**–**D**) = 40 µm). Using previously described methods [31], we quantified the epidermal thickness as indicated (**F**). *n* = 6, * *p* < 0.05, ** *p* < 0.01 vs. normal UVB (−) control groups; ## *p* < 0.01 significant amelioration vs. untreated (UVB(+)) and vehicle groups. UVB, ultraviolet B; AgNP, silver nanoparticle; TiO_2_, titanium dioxide. Data are presented as mean ± standard deviation.

Hyperplasia of the strata granulosum and spinosum have been demonstrated to be associated with histological changes in UVB-irradiated skin [31,34]. Relative to the normal controls, we observed considerable hyperplastic epidermis alterations after UVB exposure (Figure 6A vs. Figure 6B,C). By contrast, the alterations in the TiO_2_NP and AgNP groups were less severe (Figure 6D,E vs. Figure 6B,C,F), suggesting that TiO_2_NPs and AgNPs provided a shielding effect against UVB exposure.

**Figure 7:**
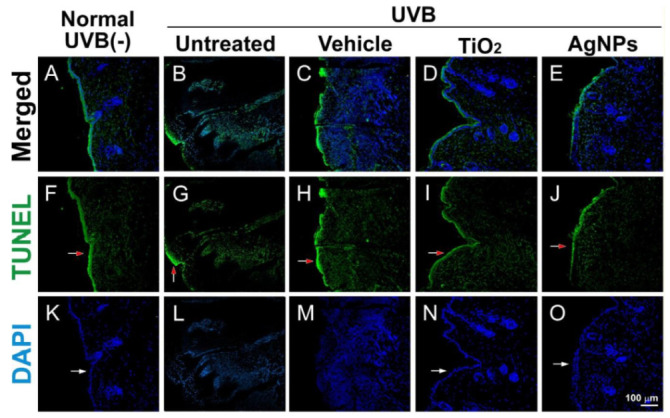
Fluorescence microscope images of mouse tissue sections. Fluorescence microscope images (**A**–**O**; merged **A**–**E**) revealed the distribution of skin cell nuclei (4′,6-diamidino-2-phenylindole (DAPI); **K**–**O**) relative to apoptotic cells (terminal deoxynucleotidyl transferase deoxyuridine triphosphate (dUTP) nick end labeling (TUNEL); **F**–**J**, red arrows indicate the green areas) in mouse tissue sections after various treatments. The margins of the stained cell nuclei (white arrows) became unclear after UVB irradiation in the UVB vehicle group (**L** and **M** vs. **K**, **N**, and **O**). Scale bar = 100 µm.

UVB-mediated skin damage was further revealed through terminal deoxynucleotidyl transferase deoxyuridine triphosphate (dUTP) nick end labeling (TUNEL) and 4′,6-diamidino-2-phenylindole (DAPI) staining (Figure 7). We found that the TUNEL signal was higher in the UVB-treated groups compared with the background staining levels in the untreated control group (Figure 7G,H vs. Figure 7F). Additionally, this increased TUNEL signal was ameliorated by TiO_2_NP and AgNP shielding (Figure 7G,H vs. Figure 7I,J). The margins of the stained cell nuclei (white arrows) became unclear after UVB irradiation in the UVB vehicle group, suggesting stronger cellular damage under conditions without shielding by NPs (Figure 7L,M vs. Figure 7K,N,O). These findings suggest that the shielding effect of AgNPs can provide effective protection against UVB irradiation.

## 3. Discussion

Silver has been used to treat burns and wounds for thousands of years [35]. Recent investigations have revealed that AgNPs are nontoxic to cultured cells in vitro [12,13,14] and are highly useful for a wide variety of applications in nanomedicine [15,16,17,18]. UV-induced pathological skin injury, such as sunburn, photoaging, and skin cancer, has been intensively investigated through cell, animal, and human studies. However, a clear demonstration of the potential of AgNP UV filters to reduce UVB-induced skin cell death and inflammation is required. In the present study, we observed that AgNPs can reduce UVB intensity, increase keratinocyte HaCaT cell survival, and ameliorate inflammatory responses of C57BL/6J mouse skin under UVB exposure. In addition, our results revealed that the efficiency of AgNPs is comparable with that of TiO_2_NPs, which are currently extensively used in sunscreens.

Bacterial infection is a problem that must be managed in patients with sunburn or thermal injury [22,23,24]. AgNPs are considered a promising antibacterial agent [36] and have long been used to treat burns [35]. AgNPs have superior physical and chemical properties over bulk silver in terms of antibacterial effect [36]. For example, AgNPs can anchor to and penetrate the bacterial cell wall. The subsequently elevated bacterial ROS levels are considered a primary step for the cytotoxic action of AgNPs because high levels of ROS have been detected in bacterial cells treated with AgNPs in various conditions [37]. This action causes physical changes in the bacterial membrane, such as membrane damage, which may lead to cellular content leakage and bacterial death [27,28,36]. In contrast to their effect on bacterial cells, AgNP treatments are not toxic to cultured cells, particularly keratinocytes, a chief component of the epidermis and a major cell type on the outermost skin layer that directly absorbs UV irradiation. Because AgNPs also exhibit absorbance in the UV range [25,26], we demonstrated that TiO_2_NPs and AgNPs considerably attenuated extreme UVB (UVI = 11) to safe levels (UVI < 2). Additionally, AgNPs efficiently protected human HaCaT keratinocytes at a density of 1 mg/cm^2^. These results suggested that AgNPs are highly suitable for use in sunscreens.

Mouse models are essential for the investigation of UV protective materials. A study revealed that, among C57BL/6J, SKH–1, and Balb/c mouse strains, C57BL/6J mice had the greatest similarity to humans in terms of susceptibility to phototoxicity, including in the thickening of the epidermis, the inflammatorily induction of TNF–α mRNA, and the accumulation of glycosaminoglycans. By contrast, hairless SKH-1 mice lacked TNF–α mRNA induction [38]. The current histological findings were similar to those of a previous report, and proinflammatory cytokine assays correlated closely with gross skin damage [31]. The overexpression of proinflammatory cytokines, namely TNF–α and IL–1β, in the skin samples of UV-irradiated C57BL/6J mice (Figure 5) was consistent with observations in UV-irradiated human skin [39]. Therefore, the C57BL/6J mouse model used in this report is suitable for evaluating UV-induced skin damage. The data presented in this report suggested that AgNPs have a protective effect on skin cells during UVB irradiation. These findings indicate that AgNPs have potential for use as a sunscreen ingredient.

## 4. Materials and Methods

### 4.1. UV-Related Equipment and Nanoparticles (NPs)

Following previously described methods, UVB irradiation was conducted using a UVB lamp (G25T8E, Sankyo Denki Co., Kanagawa, Japan) with peak emission at 306 nm. UV intensity, reported as the UVI, was measured using a UVI meter (ARCS Precision Co., Taichung, Taiwan). The conversion of UV-irradiation dose versus the UVI is shown as follows: 1 UVI = 25 *M*_W_/m^2^ UVEry (erythemally weighted UV radiation). Irradiation at a UVI of 6 for 20 min, used in our mouse experiments, were equivalent to 6 × 25 *M*_W_/m^2^ UVEry × 1200 s (=180 J/m^2^ UVEry; =1359 J/m^2^ UVB; =135.9 mJ/m^2^; =3.775 minimal erythema dose (MED); for C57BL/6J mice, 1 MED = 36 mJ/m^2^ [31,40,41]). Rutile TiO_2_NPs was purchased from Advanced Ceramics Nanotech Co., Ltd. (Tainan, Taiwan). AgNPs were purchased from Sigma-Aldrich (St. Louis, MO, USA). A UV cut-off filter was fabricated by depositing the NPs on a commercial plastic wrap film (Nan Ya Plastics Corporation, Taipei, Taiwan). A lubricating jelly (PDI, Orangeburg, NY, USA) composed of water and glycerin served as the vehicle for mixing the nanomaterials. It facilitated even dispersion of NPs and its adherence to the film and skin.

### 4.2. Human HaCaT Keratinocyte Cell Cultures

HaCaT is a human immortalized keratinocyte cell line. Following previously described methods [31], these cells were cultured with Dulbecco’s modified Eagle’s medium (DMEM) containing 10% fetal bovine serum (FBS), L-glutamine, and penicillin-streptomycin and grown in a 37 °C, 5% CO_2_ incubator. The cell culture medium was changed every two days. On confluence, the HaCaT cells were trypsinized for passage.

### 4.3. Animal Study

Wild-type male C57BL/6J mice (6–8 weeks old) were purchased from the National Laboratory Animal Center and housed in the Laboratory Animal Center, Tzu-Chi University, until they were 8–9 weeks old [31,42]. The hair on the backs of the mice was removed using a commercial hair removal cream containing thioglycolate trihydrate (approximately 250 µL/mouse) 2–3 days prior to the UVB-irradiation experiments. The mice underwent the procedures under anesthesia with an intraperitoneal injection of ketamine:xylazine (80:10 mg/kg body weight). The UVB irradiations were performed with intensity UVI 6, 20 min per day, for three cycles (135.9 mJ/cm^2^/day × 3 days) [31]. The research methods were approved by the Animal Care and Use Committee of Tzu-Chi University (approval ID 106037-A, 20 Sep 2018).

### 4.4. Measurement of UVB Shielding by Nanoparticle-Coated Films

The shielding efficiencies of NPs (average size in diameter 20, 40, and 90 nm AgNPs, and 20 nm TiO_2_NPs) in attenuating UVB radiation were investigated. The NPs were mixed thoroughly with the vehicle (lubricating jelly) and applied to thin plastic wrap films (Nan Ya Plastics Corporation, Taipei, Taiwan) at various concentrations of 0.1–4 mg/cm^2^ as UV filters. The UV intensities were measured using a UVI meter (ARCS Precision Co., Taichung, Taiwan). The UVIs of 4, 6, 9, and 11, which correspond to moderate, high, very high, and extreme exposure categories, were determined, respectively. The degree of attenuation was determined by comparing the UVI values with and without the UV filters [31].

### 4.5. Cell Viability, Cell Death, ROS, and Cytokine Analyses in HaCaT Cells

To investigate the UVB-protective effects of NPs, cultured HaCaT cells were seeded in a 96-well microplate (10^5^ cells/well) containing 200 μL/well of DMEM and 10% FBS, and grown in a 37 °C, 5 % CO_2_ incubator. 24 h later, the HaCaT cells were irradiated with UVB at a UVI of 6 with and without the UV filter containing 1 mg/cm^2^ of NPs. The culture medium was replaced by no-phenol-red medium during UVB irradiation. 24 h after UVB irradiation, the viability of cultured cells was analyzed using a cell-viability 2,3-bis-(2-methoxy-4-nitro-5-sulfophenyl)-2H-tetrazolium-5-carboxanilide (XTT) kit (Sigma-Aldrich, St. Louis, MI, USA). After incubating with XTT reagent at 37 °C for 30 min, the solutions were pipetted to a new microplate, and absorbance at 450 nm was measured using an enzyme-linked immunosorbent assay (ELISA) reader. Those cells without subjecting to UVB irradiation were considered to have 100% cell survival, and the viability of the other study groups was calculated by comparing the XTT assay results as described [31]. Pro-inflammatory cytokine TNF–α and IL–1β levels in the cell culture medium were measured using ELISA kits (BioLegend, San Diego, CA, USA). Cell labeling regents annexin V, propidium iodine (PI), and active-form caspase-3 antibody (cell death analyses) and 2′,7′–dichlorodihydrofluorescein diacetate (DCFDA) (cellular ROS analysis) were purchased from BD Biosciences (Franklin Lakes, NJ, USA). The relevant flow cytometry (FACSCalibur^TM^, Becton–Dickinson, Franklin Lakes, NJ, USA) analyses were performed following previously described methods [43,44,45,46].

### 4.6. Cytokine and Histology Examinations of Mouse Skin

The hair-removed skin of the mice (1 × 1 cm area), on which were applied with or without 1 mg/cm^2^ NPs, were irradiated with UVB at a UVI of 6 for 20 min. This procedure was repeated on three consecutive days. On the fourth day, the mice were sacrificed and the skin sections with a diameter of 0.7 cm containing the marked areas were excised for analysis. The skin samples were cut into small pieces and preserved in a 2 mL Eppendorf tube containing 700 μL of phosphate-buffered saline (PBS) and 5 mM phenylmethanesulfonyl fluoride (PMSF), which decreased cytokine degradation. The skin samples were homogenized and centrifuged at 4 °C and 16,000× *g* for 20 min. The supernatant was aspirated for additional quantification analyses of the protein and cytokine concentrations. The samples were placed on ice during processing. TNF–α, IL–1β, IL–6, and IL–10 levels in the skin, which represents the degree of inflammation, were measured using ELISA kits (BioLegend, San Diego, CA, USA), following previously described methods [31,42]. For histological examination, the skin tissues were cut into three strips, preserved in 4% formaldehyde solution, dehydrated, and embedded in paraffin wax. The tissue sectioning and hematoxylin–eosin (H&E) staining were conducted following previously described methods [31,42]. Thickness from the stratum granulosum to the stratum basale of the skin samples were measured at three random sites on each tissue strip [31]. Terminal deoxynucleotidyl transferase dUTP nick end labeling (TUNEL) and 4′,6–diamidino–2–phenylindole (DAPI) labeling were performed using regents from Thermo Fisher Scientific (Waltham, MA, USA). Fluorescence images of the skin sections were obtained using a fluorescence microscope (Nikon Eclipse E800; Nikon Taiwan, Taipei, Taiwan).

### 4.7. Statistical Analyses

The experimental results were analyzed using Microsoft Office Excel 2003, and the results reported as mean ± standard deviation (SD). Statistical significance of the obtained results was examined using a one-way analysis of variance (ANOVA) and a post hoc Bonferroni-corrected *t* test. The probability of type 1 error α = 0.05 was considered the threshold of statistical significance.

## 5. Conclusions

This study demonstrated that the shielding effect of AgNPs attenuate UVB and efficiently protects keratinocytes and C57BL/6J mouse skin from UVB-induced damage. The protective efficiency of AgNPs is comparable to that of nanosized rutile TiO_2_NPs in the mouse model. These results suggested that AgNPs have potential to serve as sunscreen ingredients.

## Supplementary Materials

Supplementary materials can be found at https://www.mdpi.com/1422-0067/21/19/7082/s1.

## Abbreviations

UVB	Ultraviolet B
UVI	UV Index
AgNPs	Silver nanoparticles
TiO_2_	Titanium dioxide
ZnO	Zinc oxide
ROS	Reactive oxygen species
TNF–α	Tumor necrosis factor–α
IL-1β	Interleukin-1β
MED	minimal erythema dose
TUNEL	Terminal deoxynucleotidyl transferase dUTP nick end labeling
