# Supplementary material for: Silver Nanoparticles Protect Skin from Ultraviolet B-Induced Damage in Mice
Source: Int J Mol Sci. 2020 Sep 25;21(19):7082. doi: 10.3390/ijms21197082 (PMC7582269; doi:10.3390/ijms21197082)
Supplement: Supplementary file 1 [file ijms-21-07082-s001.pdf]

Supporting information for:

Silver Nanoparticles Protect Skin from Ultraviolet B-Induced Damage in Mice

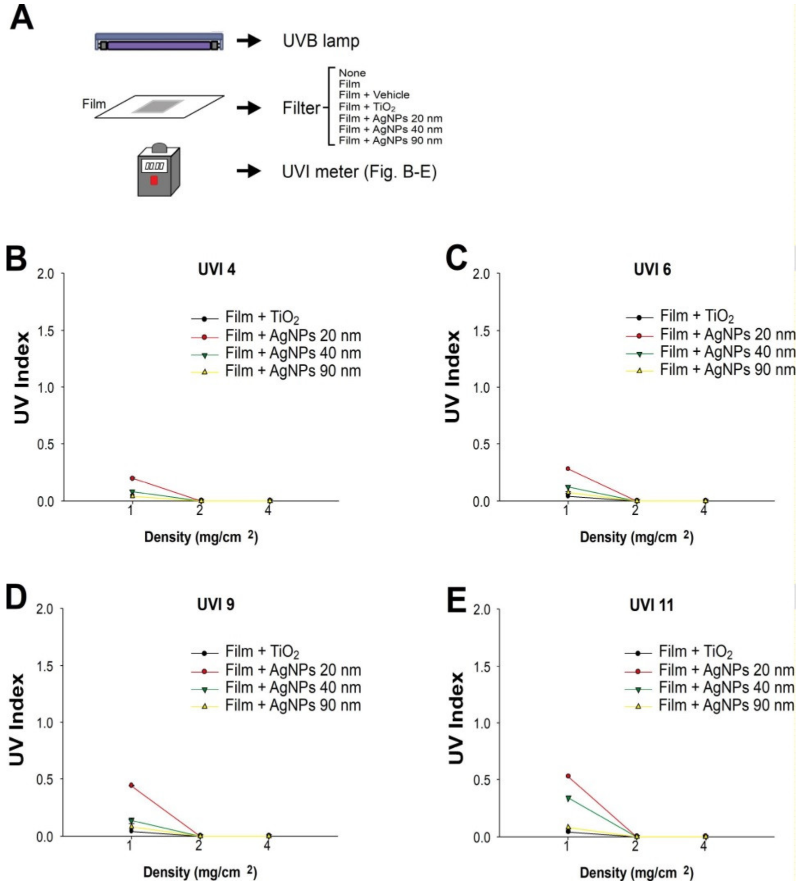

Figure 1. Relative UVB-shielding properties of tested nanoparticles (NPs). Graphs illustrated the relative UVB-shielding effects of TiO_2, and 20, 40, 90 nm AgNPs under UVB irradiations (**A**) experiment settings, with intensities of UVI 4 (**B**), 6 (**C**), 9 (**D**), 11 (**E**), respectively.

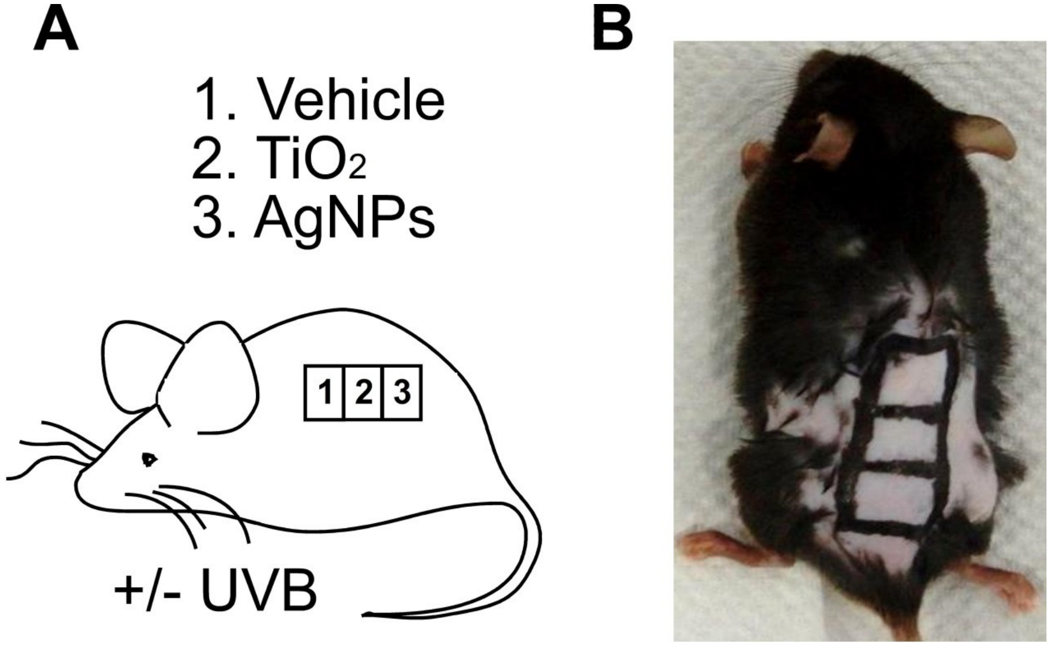

Figure 2. UVB-irradiation experiment for mice. The experiment setting (**A**) and an example of hair removed mouse used in UVB experiments (**B**).
